# ACTH signalling and adrenal development: lessons from mouse models

**DOI:** 10.1530/EC-19-0190

**Published:** 2019-06-10

**Authors:** Tatiana V Novoselova, Peter J King, Leonardo Guasti, Louise A Metherell, Adrian J L Clark, Li F Chan

**Affiliations:** 1 Centre for Endocrinology, William Harvey Research Institute, Barts and the London School of Medicine, Queen Mary University of London, London, UK

**Keywords:** ACTH, MRAP, adrenal, stem cells, MC2R

## Abstract

The melanocortin-2-receptor (MC2R), also known as the ACTH receptor, is a critical component of the hypothalamic–pituitary–adrenal axis. The importance of MC2R in adrenal physiology is exemplified by the condition familial glucocorticoid deficiency (FGD), a potentially fatal disease characterised by isolated cortisol deficiency. MC2R mutations cause ~25% of cases. The discovery of a MC2R accessory protein MRAP, mutations of which account for ~20% of FGD, has provided insight into MC2R trafficking and signalling. MRAP is a single transmembrane domain accessory protein highly expressed in the adrenal gland and essential for MC2R expression and function. Mouse models helped elucidate the action of ACTH. The *Mc2r-*knockout (*Mc2r^−^^/^^−^*) mice was the first mouse model developed to have adrenal insufficiency with deficiencies in glucocorticoid, mineralocorticoid and catecholamines. We recently reported the generation of the *Mrap^−^^/^^−^* mice which better mimics the human FGD phenotype with isolated glucocorticoid deficiency alone. The adrenal glands of adult *Mrap^−^^/^^−^* mice were grossly dysmorphic with a thickened capsule, deranged zonation and deranged WNT4/beta-catenin and sonic hedgehog (SHH) pathway signalling. Collectively, these mouse models of FGD highlight the importance of ACTH and MRAP in adrenal progenitor cell regulation, cortex maintenance and zonation.

## Hypothalamo–pituitary–adrenal axis

The hypothalamo–pituitary–adrenal (HPA) axis dictates the production of glucocorticoids secreted from the adrenal gland. Parvocellular neurosecretory neurons within the hypothalamic paraventricular nucleus (PVN) secrete corticotropin-releasing hormone (CRH) and arginine vasopressin (AVP) (1) into the hypophyseal portal circulation, and these act on anterior pituitary corticotroph cells to trigger secretion of adrenocorticotropic hormone (ACTH) (2, 3). ACTH, a 39 amino acid peptide, is produced by cleavage of its precursor protein, pro-opiomelanocortin (POMC). Other cleavage products of POMC include α-, β-, γ-melanocyte-stimulating hormones (MSH), β-endorphin, N-terminal peptide of pro-opiomelanocortin, lipotrophins and Met-enkephalin, corticotropin-like intermediate peptide (CLIP) (reviewed in 4, 5). ACTH is released into the circulation to act on peripheral sites, mainly the adrenal glands to stimulate glucocorticoid hormone production. Glucocorticoids have a negative feedback on the release of CRH and AVP at the hypothalamus and ACTH at the pituitary, thus providing tight regulation of cortisol production.

## ACTH receptor/melanocortin-2-receptor

The ACTH receptor (also known as the melanocortin-2-receptor (MC2R)), cloned in 1992 (6), is a critical component of the HPA axis and a member of the melanocortin receptor family. Other members are MC1R, MC3R, MC4R and MC5R – the functions of which were recently reviewed elsewhere (4). MC2R is unique in that it only binds ACTH, whereas MC1R, MC3R, MC4R and MC5R bind the melanocortins α-MSH,β-MSH and γ-MSH. In the adrenal gland, MC2R is expressed in all zones of the adrenal cortex. Its principal site of action is the zona fasciculata (ZF) in the generation of glucocorticoids in response to ACTH, although action on the zona glomerulosa (ZG) and zona reticularis (ZR) have been implicated in a number of physiological and disease states (7). In the case of the ZG, ACTH can acutely stimulate aldosterone production and there is now growing interest in the role of ACTH/MC2R in primary hyperaldosteronism (8, 9).

## ACTH resistance syndromes and FGD

Much of what we know about MC2R has been through the study of the ACTH resistance syndrome, FGD. FGD is a rare autosomal recessive condition clinically characterised by isolated glucocorticoid deficiency in the presence of normal mineralocorticoid function. FGD was first reported in two siblings diagnosed with Addison’s disease without hypoaldosteronism (10). Patients with FGD usually present in the neonatal period or early childhood with symptoms of hypocortisolaemia such as hypoglycaemia, failure to thrive, recurrent infections, collapse and seizures along with severe hyperpigmentation due to the extra-adrenal action of excessive plasma ACTH on MC1R in the skin melanocytes (11). Biochemically, FGD patients present with very high plasma ACTH levels often greater than 1000 pg/mL paired with very low or absent serum cortisol concentrations, hence the term ACTH resistance. Aldosterone levels are usually unaffected although derangements have been reported in a subset of patients (11, 12, 13). With the identification of additional FGD causative genes such as steroidogenic acute regulatory protein (*STAR*), minichromosome maintenance 4 (*MCM4*), nicotinamide nucleotide transhydrogenase (*NNT*), thioredoxin reductase 2 (*TXNRD2*), cytochrome p450scc (*CYP11A1*), glutathione peroxidase 1 (*GPX1*), peroxiredoxin 3 (*PRDX3*) and sphingosine 1-phosphate lyase (SGPL1), additional phenotypes including permanent or evolving mineralocorticoid deficiency have been reported, which has been recently reviewed elsewhere (14).

## Loss-of-function mutations in MC2R and FGD type 1

The first loss-of-function missense mutation in *MC2R*, S74I, was identified in 1993 after candidate-gene sequencing of two siblings with FGD (15). Following this, numerous loss-of-function mutations have been identified scattered along the whole receptor (16), cementing the importance of MC2R in HPA biology. *MC2R* gene mutations account for approximately 25% of all FGD cases (17, 18) and are referred to as FGD type I (OMIM#202200). The majority of missense mutations result in a misfolded protein and its retention in the endoplasmic reticulum while some genetic defects affect ligand binding, signal transduction or lead to a truncated protein product (4, 19). Patients harbouring *MC2R* mutations have been shown to have tall stature (16, 20, 21, 22) along with advanced or dissociated bone age (23). The mechanism for this phenomenon remains unclear but is not due to the insulin-like growth factor 1–growth hormone axis as this is normal (20). One potential explanation is that the phenotype is due to high plasma ACTH levels acting on MCRs in bone (24, 25). In support of this, ACTH has been shown to directly increase chondroprogenitor cell proliferation *in vitro* (26) and promote chondrocyte differentiation although the target MCR responsible for this action is uncertain (24). Interestingly, growth velocity falls after the introduction of glucocorticoid replacement, during which plasma ACTH levels often remain high. Another clinical feature, absent adrenarche, has also been described (27) and, in conjunction with findings that have linked polymorphisms in *MC2R* with age of onset of adrenarche, highlights the importance of ACTH in the regulation of adrenarche in children (28). The adrenal glands have been reported to be small in FGD, with histopathology showing the absence of fasciculata or reticularis cells together with disorganisation of zona granulosa (ZG) cells (16). Recent data in mouse models show that PKA signalling overactivation drives the differentiation of a reticularis-like zone in mice, supportive of the importance of ACTH in adrenal androgen regulation (29).

## Melanocortin-2-receptor accessory protein (MRAP) mutations and FGD type 2

Genetic studies of FGD patients with a normal *MC2R* identified variants in the *C21orf61* gene. These variants would result in a truncation or complete absence of the protein product, which was found to be highly expressed in the adrenal gland and adipose tissue (17). *In vitro* studies revealed that this protein interacted with MC2R and was the adrenal specific factor necessary for the transport of the receptor to the plasma membrane and for receptor signalling (17, 30, 31, 32). The protein was therefore renamed melanocortin 2 receptor accessory protein (MRAP). It is now known that mutations in *MRAP* cause approximately 20% of all FGD cases and these are termed FGD type 2 (33, 34). In comparison with FGD type 1, patients with MRAP mutations present earlier with more severe disease. Tall stature is not seen, which is probably a reflection of commencement of glucocorticoid replacement at an earlier age (21). To date, two FGD type 2 patients have been reported as requiring fludrocortisone treatment, although it is unclear if this is a transient phenomenon (35).

MRAP is a single transmembrane domain protein which is highly evolutionarily conserved in the N-terminal and transmembrane regions (17). Human MRAP has two isoforms produced by alternative splicing, MRAP-α (19 kDa) and MRAP-β (11.5 kDa), which differ in the C-terminus. The functional difference between the two isoforms is unclear although differences have been reported in ACTH-binding capacity and cAMP generation (36). Interestingly, MRAP can form unique antiparallel transmembrane homodimers, which together with its paralogue MRAP2 are as yet the only group of proteins known to do so in eukaryotic cells. These MRAP homodimers form multimers with MC2R (37, 38, 39). The orientation of the dimers is critical to function (37, 38, 39); moreover, these antiparallel homodimers are made early on in the biogenesis of the protein, are maintained and stable, and in a heterologous cell based system have a half-life of 2 h (40). Although MRAP *in vitro* binds and modulates the function of other MCRs (30), it is unclear what physiological relevance this has at present.

## Mouse models of FGD

The first mouse model of FGD was generated by Chida *et al*. in 2007 (41). The *Mc2r* KO mouse model was the last member of the melanocortin receptor family to be knocked out in mice. We recently generated a novel *Mrap* KO mouse model which, like FGD, has isolated glucocorticoid deficiency. Both models are discussed in more detail below.

### *Mc2r*-knockout mouse model

A mouse model of MC2R deficiency was generated by replacing the whole coding region of *Mc2r* with a neomycin-resistance gene cassette (41), leading to complete absence of *Mc2r* transcript in *Mc2r^−^^/^^−^* mice. On a C57BL/6J background, the majority of *Mc2r^−^^/^^−^* mice did not survive 48 h after birth. Genotyping of 129 living mice at 4 weeks of age identified that only 9 (7%) were *Mc2r^−^^/^^−^*. It is worth noting that on a B6/Balbc mix background approximately half *Mc2r^−^^/^^−^* mice survived to adulthood, suggesting genetic modifiers at play (42). Detailed analysis demonstrated that *Mc2r^−^^/^^−^* mice failed to produce glucocorticoids and developed profound neonatal hypoglycaemia resulting in high mortality levels during the first days of life. Moreover, pups born to homozygous *Mc2r*-null parents died before postnatal day 0.5 due to lung failure, highlighting the importance of glucocorticoids in foetal lung maturation (41). In addition to glucocorticoid deficiency, *Mc2r^−^^/^^−^* mice also had significantly lower serum levels of aldosterone and catecholamines (41). Epinephrine levels were significantly reduced in *Mc2r^−^^/^^−^* animals, whilst dopamine and norepinephrine concentrations were unchanged. Examination of the adrenals of surviving adult mice (never replaced with glucocorticoids) revealed dysmorphic glands with gross hypoplasia of the ZF. Moreover, lipid droplets within the ZF cells were markedly reduced or absent, but cell nuclei were normal. However, the precise cell identity of such ‘ZF’ cells is not known. Interestingly, the adrenal capsule in the *Mc2r*-null mice was thickened compared to *Mc2r^+/+ ^*littermates. The majority of naturally occurring MC2R loss-of-function mutations are missense mutations, many with residual function (4, 19). Location and type of reported MC2R mutations are summarised in a recent review (4). As a demonstration of this, patients with missense MC2R mutations have been known to present later on in childhood (21). Several more severe cases including homozygous nonsense or frame shift mutations in MC2R have been described to have hyponatraemia and/or disruption of renin-angiotensin-aldosterone system (12, 13, 35). The majority of these are transient though permanent fludrocortisone replacement has been described in several children (35). Hence, although the complete KO of Mc2r in mice is not fully representative of human FGD type 1, the model has nevertheless highlighted crucial actions of ACTH and/or glucocorticoids during development.

### *Mrap*-knockout mouse model

We recently reported a *Mrap* KO (*Mrap^−^^/^^−^)* mouse model created by targeting the first coding exon of *Mrap,* which led to complete absence of transcript and protein in homozygote mice (43). Intercrossing heterozygote (*Mrap^+/^^−^*), also on a C57BL/6J background, demonstrated high neonatal mortality. Out of 325 mice generated from breeding *Mrap^+/^^−^* mice, only three *Mrap^−^^/^^−^* mice (<1%) survived until weaning (43). The new-born pups born from heterozygous parents with normal adrenal function died before postnatal day 1 and morphologically showed immature lungs and lack of hepatic glycogen stores (43). Glucocorticoid treatment of pregnant *Mrap^+/^^−^* dams rescued the phenotype, resulting in *Mrap^−^^/^^−^* mice born at the expected ratio of 25%. This indicated that the observed high mortality in homozygous *Mrap^−^^/^^−^* newborns was likely to be due to glucocorticoid deficiency rather than a direct effect of *Mrap* deletion. Moreover, this phenotype highlights the importance of foetal rather than maternal glucocorticoids in pre-partum neonatal adaptation in the *Mrap^−^^/^^−^* mouse.

The severity of this phenotype is consistent with the human data whereby FGD type 2 patients present soon after birth (21), thought to be due to the severity of the mutations in MRAP that lead to complete disruption or absence of protein (21). Naturally occurring mutations in MRAP (location and type) have recently been reviewed (4).

Adult *Mrap^−^^/^^−^* mice of both genders exhibited a grossly dysmorphic adrenal cortex with the glucocorticoid synthesis pathway severely downregulated and unable to produce glucocorticoids in the presence of high plasma ACTH levels. Surprisingly, unlike the *Mc2r^−^^/^^−^* mice, circulating aldosterone and catecholamine levels were unaffected (41, 43). The enzyme phenylethanolamine N-methyltransferase (PNMT), which is responsible for conversion of norepinephrine to epinephrine, appeared to be unaffected in *Mrap^−^^/^^−^* mice even though PNMT expression is known to be dependent on glucocorticoid action. In contrast, PNMT is markedly reduced in *Mc2r^−^^/^^−^* (41). As both knockout models are on a C57BL/6J background, one obvious difference is that the *Mrap^−^^/^^−^* mice received relatively high doses of glucocorticoids between E17.5 until weaning. This could contribute to the discrepancy in PNMT between *Mc2r^−^^/^^−^* and *Mrap^−^^/^^−^* mice, especially in light of the fact that prenatal exposure to glucocorticoids has been shown to lead to increased PNMT mRNA by RT-PCR and elevated plasma epinephrine in adult rats (44). The discrepancy between aldosterone levels in *Mc2r^−^^/^^−^* and *Mrap^−^^/^^−^* mice could be due to a number of possibilities, such as *Mrap*-independent aldosterone production (although *in vitro* the MC2R is non-functional in the absence of MRAP and MRAP protein expression closely mirrors that of MC2R in the ZG), differences in salt intake between the two models (both rodent lines are fed *ad libitum* on a standard chow diet although, the salt content may differ) and finally alterations of the renin–angiotensin–aldosterone system have also been reported in animals exposed to glucocorticoids prenatally (45). Determining the mechanism of such differences would further dissect adrenal ACTH action. Importantly, however, the absence of mineralocorticoid and catecholamine deficiency in *Mrap^−^^/^^−^* mice makes it a unique model for studying FGD and isolated glucocorticoid deficiency.

Similar to the *Mc2r^−^^/^^−^* mouse model, the *Mrap^−^^/^^−^* adrenal capsule is thickened, which in the *Mrap^−^^/^^−^* mice was shown to be due to an increased cell number, rather than hyperplasia. This is of particular interest as the adrenal capsule and the subcapsular region are known to contain adrenocortical stem/progenitor cells capable of dividing, migrating centripetally and differentiating into mature steroid-producing cell types (46, 47, 48). In keeping with this, the absence of MRAP and therefore ACTH signalling resulted in small adrenals with grossly deranged cortex zonation (discussed in subsequent sections).

### Other MRAP mouse models: transgenic MRAP adipose tissue overexpression mouse model

Prior to its renaming in 2005, MRAP was first identified as a putative novel membrane protein selectively expressed during adipogenic conversion of 3T3-L1 cells and called FALP (fat tissue-specific low molecular weight protein) (49). The protein expressed in both brown and white fat tissue and highly expressed during adipogenesis (49, 50). More recently, the importance of MRAP in energy balance was demonstrated using transgenic mice overexpressing MRAP in a fat-specific manner, under the control of the aP2 (adipocyte fatty acid-binding protein) promoter (51). These mice were shown to be protected against diet-induced obesity and diabetes, through enhancement of ACTH-induced lipolysis. Therefore, this demonstrated for the first time the emerging importance of MRAP in metabolism.

## ACTH and adrenocortical renewal and zonation – contribution of *Mrap* and *Mc2r* KO mouse models

### Adrenocortical renewal and regeneration

The adrenal gland has a great capacity to respond to changes, renew and regenerate. For example, activation of the HPA axis leads to expansion of the ZF and increased expression of CYP11B1 and glucocorticoid production, whilst suppression by dexamethasone leads to the contraction/atrophy of the ZF, and reduced CYP11B1 and glucocorticoid production (52). This is not specific to the ZF and activation or inhibition of the renin–angiotensin–aldosterone system (i.e. triggered by a diet low in sodium or treatment with ACE inhibitors, respectively) results in similar changes in ZG morphology, CYP11B2 expression and aldosterone production (53). This together with the appearance of compensatory growth of the contralateral adrenal gland following unilateral adrenalectomy (54) demonstrates the dynamic nature of the adrenal gland. Apart from remodelling, the adrenal gland can also regenerate from residual adrenal capsular and adherent ZG cells in enucleation studies (55, 56). Regeneration arises from cell proliferation and differentiation and is associated with a thickened adrenal cortex (57).

### Role of ACTH in adrenal progenitor cell differentiation and maintenance

ACTH administration can induce ZF hyperplasia, with no effect on the ZG (58). More recently, the localisation of *Mc2r* and *Mrap* to the undifferentiated zone (layer of cyp11b1/b2 negative cells located between the ZG and ZF) in the rat adrenal (59, 60), suggested a role for ACTH in the differentiation of progenitor cells towards the ZF phenotype *in vivo*. However, the data from remodelling experiments provides somewhat conflicting evidence for the exact role of ACTH in adrenal progenitor cell differentiation and maintenance. For example, some studies show that treatment with dexamethasone blocks ZF proliferation and compensatory growth of the contralateral gland following unilateral adrenalectomy (54, 61), whilst other studies do not show this (62, 63). Hypophysectomy, with the complete removal of the pituitary gland, blocks adrenal gland regeneration following enucleation (64) but does not completely block compensatory growth (52, 65), and ACTH has even been shown to inhibit this process (65). However, overall there is agreement that hypophysectomy reduces the extent of compensatory growth suggesting the presence of a pituitary factor. One possible factor is pituitary-derived N-POMC, derived from the POMC cleavage product pro-γ-MSH. Neutralising antibodies against pro-γ-MSH inhibit both adrenal regeneration and compensatory growth. However, pro-γ-MSH has no direct mitogenic activity (66); hence, it has been suggested that further processing of pro-γ-MSH to peptides such as N-POMC with mitogenic activity is required (67). Evidence of the need for pituitary factors, including ACTH, comes from work on *POMC^−^^/^^−^* animals that have complete absence of the POMC peptide (68, 69). *POMC^−^^/^^−^* mice have adrenal glands that fail to proliferate postnatally leading to atrophic adrenals which become undetectable with age (70). These adrenal glands from *POMC^−^^/^^−^* animals can be rescued in size and function, following transplantation into a WT recipient animal with physiological levels of all POMC peptides. Interestingly, N-POMC cannot restore adrenal growth in POMC-null mice (71). However, high-dose replacement of ACTH over several days restores *POMC^−^^/^^−^* adrenal weight, morphology and corticosterone secretion (68), but it has been suggested this is due to hypertrophy of the ZF rather than full regeneration of the adrenal gland (72).

### Contribution of *Mc2r* and *Mrap* KO mouse models to understanding of adrenal stem/progenitor cell differentiation and maintenance

The MC2R and MRAP KO mouse models add to our existing knowledge of the role of ACTH in adrenal gland stem/progenitor cell differentiation and gland maintenance. Assessment of the adrenal glands of *Mrap^−^^/^^−^* mice at embryonic day 17.5 shows that the adrenal sizes are comparable to *Mrap^+/+ ^*mice, derived from intercrosses of heterozygous mice with normal HPA activity. However, when assessed at 8 weeks of age the gland is significantly reduced in size with a thickened adrenal capsule (43). One major issue is the separation of the effects of absent ACTH action and glucocorticoid deficiency that are both present in adult *Mrap^−^^/^^−^* mice. After lifetime glucocorticoid replacement the capsule reduced in thickness but still remained significantly increased compared to wild-type littermate animals suggesting that both glucocorticoid deficiency and absent ACTH action contribute to the expansion of the stem cell niche in the adrenal gland, in keeping with some of the data described above. Treatment of WT control mice with glucocorticoid resulted in biochemically undetectable plasma ACTH coupled with corticosterone within the normal range. These mice have a thickened capsule and thus taken together this is highly suggestive that the absence of ACTH action is a key contributor to capsule thickening.

The *Mrap^−^^/^^−^* mouse model also introduces some novel concepts in the growing landscape of molecular pathways and factors involved in adrenal stem/progenitor cell determination and differentiation. In fact this field is rapidly gaining pace with the identification of new factors required for the adrenal homeostasis and zonation such as RSPO3, EZH2 and ZNRF3 (73, 74, 75) and (reviewed in 47, 53). The WNT4/β-catenin and SHH pathways have been described to be key drivers of ZG identity (76).

Cells that express WNT/β-catenin, which co-express SHH and CYP11B2, normally reside in the subcapsular region (41, 42, 43). It has been shown that cAMP/PKA, activated by ACTH signalling in the adrenal gland, represses the WNT/beta-catenin pathway to allow lineage conversion and correct adrenal cortex zonation (46). Consistent with this is the absence of ZF in the adrenals of *Mrap^−^^/^^−^* mice, whilst ZG is intact and functional. However, the *Mrap^−^^/^^−^* mice identifies a concentric zone of cells between the ZG and adrenal medulla that are negative for Cyp11b2, Cyp11b1 and 20-αHSD and hence not terminally differentiated ZG, ZF or ZR/foetal X-zone cells respectively. These cells are WNT4/β-catenin positive, but negative for the downstream canonical targets of WNT signalling (LEF1 and DAB2), suggesting that canonical WNT signalling is not active in these cells. Another option is that these cells were once LEF1- and DAB2-positive and have subsequently lost some of ZG features. The precise origin, fate and signalling potential of these cells are currently being investigated.

**Figure 1:**
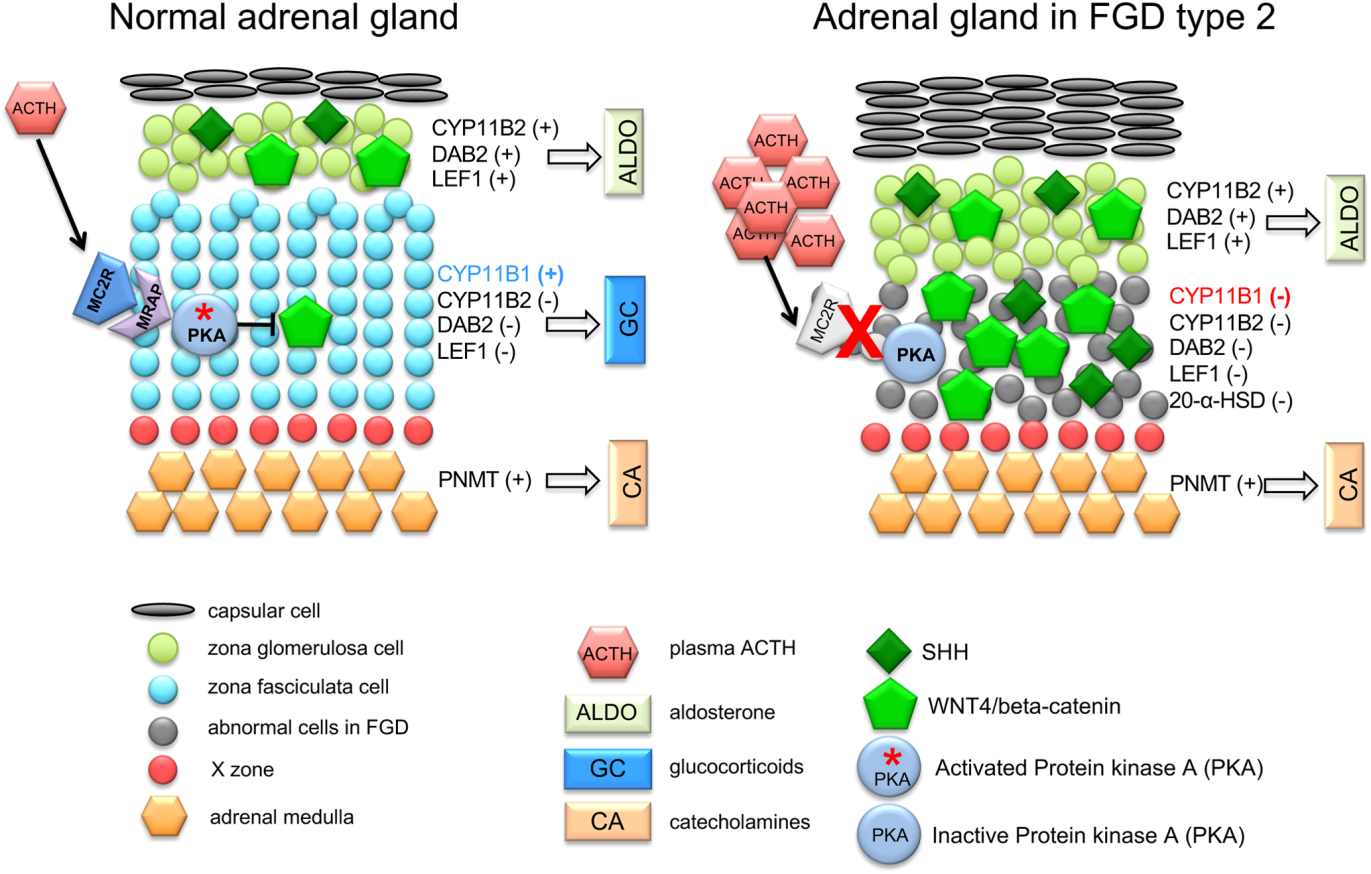
Model illustrating the morphological alterations and pathway deregulation in the adrenal gland of FGD type 2. In the normal adrenal cortex, activation of SHH and WNT4 signalling results in driving the expression of CYP11B2, DAB2 and LEF1 leading to a zona glomerulosa (ZG) lineage. Activation of PKA due to the action of ACTH on MC2R/MRAP complex suppresses WNT4 resulting in inhibition of CYP11B2, DAB2 and LEF1 and differentiation into ZF cells. In FGD type 2 (*Mrap^−^^/^^−^* mice) adrenal glands, the lack of PKA activation results in complete absence of ZF and WNT4/SHH accumulation outside the ZG, where WNT4 expression in such cells do not lead to a functional ZG identity. Morphologically the ZG is expanded and is able to secrete aldosterone. The adrenal medulla in FGD type 2 is morphologically intact and secretes both epinephrine and norepinephrine.

SHH signalling is another key pathway regulating the differentiation of progenitor cells into steroidogenic cells (77, 78). SHH-positive cells in the stem cell niche, which are located in the subcapsular region in mice and in the undifferentiated zone in rats, have been shown through lineage tracing to differentiate into all cortical cell populations in mice. The majority of SHH-descendants become ZG cells first and then transition to ZF cells during centripetal migration (48, 77). In SHH KO mice, the adrenal capsule is reduced to a single cell layer, suggesting that SHH could act as a capsule cell mitogen/chemoattractant for noncapsule mesenchymal cells or to maintain capsule progenitors (77). In the absence of MRAP, we see thickened capsule, upregulated SHH expression and ectopic SHH expression throughout the cortex, no longer restricted to the subscapular region. Together with the co-expression of WNT4 and CYP11B2, this is suggestive that these cells have acquired ZG features. Glucocorticoid treatment only partially attenuated this phenotype. Interestingly, the levels of Gli1, which is a canonical target of SHH, were unchanged despite high SHH expression. Determining the reason for this dissociation between high SHH and Gli1 which is unchanged as well as the co-ordination with other factors involved with capsule thickness such as FGF signalling will be investigated in the future (Fig. 1).

In conclusion, the mouse models of FGD have helped elucidate the action of ACTH and glucocorticoids in adrenal development, progenitor cell renewal and zonation. Further studies will focus on the role of ACTH signalling in physiology as well as how this can be altered in disease states.

## Declaration of interest

The authors declare that there is no conflict of interest that could be perceived as prejudicing the impartiality of this review.

## Funding

The work reviewed was funded by The Medical Research Council UK (MRC/Academy of Medical Sciences Clinician Scientist Fellowship Grant G0802796 to L F Chan).
